# Characterization of a Small Auxin-Up RNA (SAUR)-Like Gene Involved in Arabidopsis thaliana Development

**DOI:** 10.1371/journal.pone.0082596

**Published:** 2013-11-27

**Authors:** Marios Nektarios Markakis, Agnieszka Karolina Boron, Bram Van Loock, Kumud Saini, Susanna Cirera, Jean-Pierre Verbelen, Kris Vissenberg

**Affiliations:** 1 Department of Biology, University of Antwerp, Antwerpen, Belgium; 2 Department of Animal and Veterinary Basic Sciences, University of Copenhagen, Frederiksberg C, Denmark; Wake Forest University, United States of America

## Abstract

The root of Arabidopsis thaliana is used as a model system to unravel the molecular nature of cell elongation and its arrest. From a micro-array performed on roots that were treated with aminocyclopropane-1-carboxylic acid (ACC), the precursor of ethylene, a Small auxin-up RNA (SAUR)-like gene was found to be up regulated. As it appeared as the 76th gene in the family, it was named SAUR76. Root and leaf growth of overexpression lines ectopically expressing SAUR76 indicated the possible involvement of the gene in the division process. Using promoter::GUS and GFP lines strong expression was seen in endodermal and pericycle cells at the end of the elongation zone and during several stages of lateral root primordia development. ACC and IAA/NAA were able to induce a strong up regulation of the gene and changed the expression towards cortical and even epidermal cells at the beginning of the elongation zone. Confirmation of this up regulation of expression was delivered using qPCR, which also indicated that the expression quickly returned to normal levels when the inducing IAA-stimulus was removed, a behaviour also seen in other SAUR genes. Furthermore, confocal analysis of protein-GFP fusions localized the protein in the nucleus, cytoplasm and plasma membrane. SAUR76 expression was quantified in several mutants in ethylene and auxin-related pathways, which led to the conclusion that the expression of SAUR76 is mainly regulated by the increase in auxin that results from the addition of ACC, rather than by ACC itself.

## Introduction

Plant growth, development and responses to biotic and abiotic stimuli are largely regulated by plant hormones [1]. Besides the five well known phytohormones auxin, ethylene, cytokinin, abscisic acid and gibberellins, several other molecules including brassinosteroids, salicylic acid, jasmonate and relatively recently identified molecules like strigolactones [2] are considered as phytohormones [3,4]. The use of plant hormones allows the plant to coordinate plant behaviour by regulating transcription or by modulating protein activity. This is usually achieved by generating specific differences in the concentrations of a relatively small set of hormones at a well-defined spatial and temporal scale [5–8].

One of the major plant hormones, ethylene, has been shown to be involved in several aspects throughout plant development, such as in the release of seed germination [9], seedling growth [10], adventitious root formation [11,12], root hair growth [12,13], flower induction in Bromeliads [14], induction of femaleness in dioecious flowers [15], stimulation of senescence of leaves and flowers [16], abscission of leaves and fruit [17], stimulation of fruit ripening in climacteric species [18] and in stress responses including biotic (pathogens) as well as abiotic stresses (cold stress, hypoxia and wounding) [19]. From the above-mentioned processes it is clear that ethylene is a principal actor exerting its effects throughout the whole plant life. 

The biosynthetic pathway of ethylene starts at the amino acid methionine, which is converted by the enzyme S-adenyl-methionine synthase to S-adenyl-methionine (S-AdoMet). This in turn is converted to ACC by ACC synthase and subsequently to ethylene by ACC oxidase (for review 20–22). Besides the well-studied triple response in dark-grown hypocotyls ethylene also has a marked effect on root growth in light-grown plants. Le and others [23] have shown that treatment of Arabidopsis roots with ethylene, supplied as its precursor ACC, results in a fast and concentration-dependent inhibition of root elongation, suggesting that this hormone could act as a natural modulator of root elongation control. How exactly it exerts its control on elongation is not completely clear at the moment, but evidence points to a control involving symplastic as well as apoplastic events [24,25].

To reveal which genes and processes are responsible for the ethylene-induced cell elongation arrest in the Arabidopsis root a CATMA microarray analysis on control and 3 hours ACC-treated roots was performed. Statistical analysis of the micro array data identified 240 differentially expressed genes [26]. When the function of these differentially expressed genes was investigated, it appeared that the majority of the genes have an unknown function. The second largest group of genes contained genes with (presumed) functions in cell wall biosynthesis and metabolism followed by transcriptional and translational regulation, and stress-induced genes. It was shown before that ethylene treatment of roots results in changes in auxin transport and/or biosynthesis [27–30]. It is therefore no surprise that in the microarray analysis several ethylene-related and auxin-related genes were identified [26], providing again evidence for ethylene/auxin crosstalk, whether primary (which occurs by activation of genes that contain auxin and ethylene responsive elements in their promotor) or secondary (by activation of genes that regulate the other hormones' synthesis, transport, signalling or response) (for review see 31).

It is known that three gene families are rapidly and transiently induced in response to auxin: the auxin/indoleacetic acid (Aux/IAA), Gretchenhagen-3 (GH3) and the Small auxin-up RNA (SAUR) family [32]. SAUR genes, which currently count 82 members together with SAUR-like genes on the TAIR web site (www.arabidopsis.org), are originally characterized in soybean. Although the biochemical or developmental functions of this family remains largely unknown, members have been reported to be membrane and/or cytoplasm-localised [33,34], to accumulate within 2.5 minutes after auxin treatment [35,36], to be correlated with elongating tissues [33,34,36–38] and to negatively influence synthesis of auxin and proteins for polar auxin transport [39,40]. 

In this manuscript the aim was to identify the role of the fifth most up regulated gene in Arabidopsis roots treated for 3 hours with ACC, identified by the microarray analysis described in [26]. This gene was At5g20820 and it codes for an auxin-responsive protein-related protein with sequence similarity to SAUR proteins. This gene was characterized and its role in elongation control was investigated. 

## Materials and Methods

### Plant Materials, Growth Conditions and Phenotype Experiments

Wild type seeds (Col-0) and a SAUR76 (At5g20820) knock-out line (N307666) were obtained from the Arabidopsis stock centre (NASC, Loughborough, Leistershire, UK). All plants were grown in an environmentally controlled growth chamber (15 photons/cm/s; 16h light/8h dark; 24°C). Seeds of wild type, knock-out and over expression lines were grown in vivo in Tref Substrate soil in the same tray and under the same conditions to bulk seeds of the same age for the phenotype experiments. For root measurements and measurements of gene expression levels *in vitro* seedlings were used. Seeds were surface-sterilised for 5 minutes in 6% commercial bleach followed by 3 rinses in EtOH. Seeds were placed on 1/2 MS plates containing 2.2g/l Murashige and Skoog [41] salts including vitamins (Duchefa, the Netherlands), 1% (w/v) sucrose (Duchefa), adjusted to pH 5.7 [KOH] and solidified with plant agar 8g/l (Duchefa). Plates were closed with one revolution of laboratory film (Parafilm, Pechiney Plastic Packaging, Menasha, WI, USA) and subsequently stratified for 2 days at 4°C in the dark. Plates were then placed in a growth chamber (15 photons/cm/s; 16h light/8h dark; 24°C).

### Phylogenetic analysis

To elucidate the relation of SAUR76 with other SAUR and SAUR-like proteins, a phylogenetic tree was created from parsimony analysis. The protein sequence of SAUR was used to retrieve related sequences from the BLAST program [42,43]. These retrieved sequences were aligned in ClustalW 1.87 and a phylogenetic tree was made from neighbor-joining analysis using Mega 5.05 software [43]. The bootstrap values are shown in the tree and 1000 replicates were used.

### Generation of Constructs

All oligonucleotides were obtained from Eurogentech (Seraing, Belgium) and all constructs were made using Gateway technology (Invitrogen, Carlsbad, CA, USA; [44]). The 2.1 kb promoter region of At5g20820 used for the promoter::reporter construct was amplified from genomic DNA using Pfu DNA polymerase (Promega Corp., Madison, WI, USA) and the oligonucleotides prom20820FOR (5’-GGGGACAAGTTTGTACAAAAAAGCAGGCTTAAC CCTTGTGTCAGAGTTATAATCC-3’) and prom20820REV (5’-GGGGACCACTTTGTACAAGAAAGCTGGGTTGTCGAAGAGAGAAAGG AGAATG -3’) containing the Gateway-compatible AttB recombination sites. The ORF for the overexpression and N-terminal GFP fusion line was isolated using the BAC clone T1M15 ordered from ABRC (Arabidopsis Biological Resource Centre) and the oligonucleotides Fattb1at5g20820 (5’-GGGGACAAGTTTGTACAAAAAAGCAGGCTT CATGGCGAAAGGAGGAAAC-3’) and Rattbat5g20820-N (5’- GGGGACCACTTTGTACAAG AAAGCTGGGTTTTAACAAGCGTAGAACTCG-3’) containing the AttB recombination sites. For the C-terminal GFP-fusion construct, the reverse primer was constructed to exclude the stop-codon Rattbat5g20820-C (5’-GGGGACCACTTTGTACAAGAAAGCTGG GTTTCAAGCGTAGAACTCG-3’). The amplified promoter and open reading frames were first recombined with pDONR207 (Invitrogen) and the resulting clones were checked by colony PCR and subsequently sequenced at the Sequencing Facility of the VIB (Flemish Institute of Biotechnology). Clones found to have a 100% correct sequence were subsequently recombined with the destination vectors. For promoter::GUS or GFP constructs pGWB3 and pGWB4 [45] were used respectively, for CaMV 35S-driven overexpression pGWB2, for N and C-terminal GFP fusion pGWB5 and pGWB6 respectively [45] was used. The resulting clones were subsequently checked by colony PCR and then used to electroporate Agrobacterium tumefaciens C58Rif harbouring pMP90 [46]. Plant transformation of wild type (Col-0) was carried out by flower dip basically as described in [47] using a transformation buffer containing 5% sucrose, MgCl_2_6H_2_0 (4mM) and 0.02% (v/v) Silwet L-77 (polyalkyleneoxide modified heptomethyltrisiloxane) (GE Specialty Materials, Geneva, Switserland). For every transgenic line, more than 21 independent lines were selected and approximately 4-7 lines were brought to the homozygous state in order to be used in further experiments.

### Confocal Microscopy

GFP was localised with a Nikon C1 laser scanning confocal unit (D-Eclipse-C1, Nikon, Melville, NY) equipped with an argon and a helium/neon laser line fitted onto an upright microscope (Eclipse E600, Nikon, Melville NY) in combination with a 10x planfluor (NA: 0.30), 20x planfluor (NA: 0.50), 40x planfluor (NA: 0.95) or 60x planfluor (NA: 01.95) objective manufactured by Nikon (Melville, NY). For easy identification of cell types the cell walls were counterstained for less than 1 minute in an aqueous 0.1 mg/ml propidium iodide (Across Organics) solution after which the seedlings were rinsed briefly in distilled water before being mounted in distilled water.

### GUS Staining

For GUS histochemical analysis, seedlings were grown in 1/2 MS liquid medium with similar composition as described above, but without plant agar. Approximately 10 to 15 sterilised seeds were added to 5 mL of liquid medium and placed directly on a rotary shaker (INFORS-HT TR-225) at 80 revolutions per minute located in an environmentally controlled growth chamber. GUS staining was performed according to a modified protocol of [48]. In brief, plant tissues were submerged in a staining solution (potassium phosphate buffer 200mM [pH 7.0], potassium ferrocyanide 1mM, potassium ferricyanide 1mM, disodium EDTA 10mM, 5-bromo-4-chloro-3-indolyl-β-D-glucuronide 1mM) and incubated for maximum 4 hours at 37°C. After staining, tissues were rinsed 3x10 minutes in H2O and subsequently fixed in ethanol:acetic acid (3:1 v/v) for at least 48h and cleared in 8M NaOH for approximately 1h. Images were taken in bright field mode using a Zeiss Axioskope (Zeiss, Jena, Austria) equipped with a Nikon DXM 1200 or Nikon DS-Fi1 digital camera (Nikon Instruments Inc., Melville, NY, USA). 

### Isolation of a Homozygous Knock-out Line for At5g20820

Heterozygous seeds of the line N307666 were obtained from NASC and sown on soil. Genomic DNA was extracted according to a modified protocol of [49]. Homozygous, heterozygous and wild type plants were identified using genomic primers GABI_591E03.FP(5’-CCGAATCGGTAATGGCGAAAGGAGG-3’) and GABI_591E03.RP (5’- GCGTAGAACTCG ACAAGTTCGTAGACCG -3’) flanking the T-DNA insertion site and T-DNA specific primers T-DNA.GK/N30766 (5’-ATATTGACCAT CATACTC ATTGC-3’). The level of the At5g20820 transcript was semi-quantitatively determined by preparing cDNA using the SuperScript™ II Reverse Transcriptase (Life Technologies) from homozygous At5g20820 T-DNA insertion plants and from wild type plants. The PCR product amplified by cDNA-specific primers was analysed by 1.5% agarose gelelectrophoresis. In addition the expression level in the homozygous knock-out line was confirmed by qRT-PCR analysis with 2 endogenous controls using TaqMan probes At02302302_s1 At02270958_gH and At02337969_g1 for At5g20820, Actin 8 and EF-1-alpha respectably.

### RNA Isolation and cDNA Synthesis

For RNA isolation different methods were used depending on the nature of the experiments. In general, for plant material that is easily accessible the TRIzol Reagent (Life Technologies) was used according to the guidelines of the manufacturer. For a limited amount of samples, such as dissected root elongation zones, the RNAqueous-Micro Kit with Plant RNA Reagent (Life Technologies) was used due to its ability to isolate RNA from 1-5 mg of materials. The nanodrop (ND1000) was used for the quality control of the isolated RNA. The cDNA synthesis was performed with SuperScript TM II Reverse Transcriptase (Life Technologies) according to guidelines provided in the protocol. In addition to that, the samples were treated with Deoxyribonuclease I in order to avoid genomic DNA (since the target was a single exon gene). 

### Identification of Overexpression and GFP-Fusion Lines

Furthermore, equal amount RNA of the selected homozygous over expression lines was used to create cDNA, which was then used to run a PCR with gene-specific primers. A semi-quantitative approach was used to compare the expression in the selected line against the WT. In more detail, equal amount of cDNA from 5-6-day-old plants grown on solid 1/2 MS medium was used to run a PCR and 10 micro liters were taken from the PCR-solution during cycle 25, 28, 31 and 34 and analysed by gel electrophoresis. Furthermore, once the lines with a clear overexpression were identified, qPCR analysis was performed to quantify the expression level. 

### qPCR Analysis

For the qPCRs done in this work two methods were used. The analysis of the expression levels of At5g20820 in Wild type (Col-0) roots and leaves was done using the Quantifast SYBRgGreen Master mix from Qiagen with primers qPCR-At5g20820-forw (5’- CCGCTCTTCCAGCAGCTA-3’) and qPCR-At5g20820-rev (5’-AACTTCGCACGACACAGAGA T-3’), and primer sets to amplify 3 internal reference genes 18S, ACT8 and EF1alpha. Quantification of expression levels in all other experiments was done using the TaqMan Universal Master Mix II with UNG (Life Technologies). With 1:8 diluted cDNA samples multiplex PCRs were performed with ACT8 (id. At02270958_gH) as endogenous control and TaqMan probe (id At02302302_s1) corresponding to At5g20820. For all reactions at least 3 biological repetitions were performed. 

### Western Blot Analysis

Western blot analysis was performed on the protein-GFP fusion lines to detect the size of the GFP-containing proteins. Protein isolation was performed as described in [50] and isolated proteins were separated on a pre-casted NuPage 4-12% Bis Tris Mini gel with MOPS buffer (Life technologies). Proteins were then blotted to a nitrocellulose blotting membrane. Non-specific antibody binding sites were blocked with 5 % BSA in TBS-T before the rabbit anti-GFP primary antibody was added at a concentration of 0.1-10 μg/mL. After a wash the goat anti-rabbit secondary antibody conjugated with HRP was added at a concentration of 1-1000 μg/mL and used to visualize the primary antibody after incubation with DAB Peroxidase Substrate Solution according to the manufacturer's protocol (Life Technologies). 

### Phenotype Experiments

For root length measurements seedlings were grown in vitro as described above on solid 1/2MS medium before being photographed with a Canon Eos 50D digital camera (Canon, Japan). Measurements were performed using the freely available tool ImageJ (http://rsbweb.nih.gov/ij/; [51]).

Leaf and cell measurements were performed as described in [52]. Seedlings were grown in vitro as described above on solid 1/2MS medium. The rosette area was measured on day 22 and the area of leaf series was measured on day 25 after stratification in 3 replicate experiments using ImageJ. Based on the leaf series data, leaf 4 was used for further cell analysis. Leaves were placed in 70% ethanol overnight to remove chlorophyll before being transferred to lactic acid for clearing. Using DIC microscopy (Nikon AZ100 Multizoom equipped with a Nikon DS-Ri1 digital camera) epidermal cells of the abaxial leaf side were visualised and hand-drawn on an LCD drawing tablet (Wacom drawing pad). Images were converted to high resolution (2556x2045) and analysed with linux-based image analysis software [53]. Calculations were made as described in [52]. 

### Statistical analysis

The Student's t-test was used to compare measurements of root length, leaf parameters and expression values using qPCR. A p-value of <0.05 was considered to be statistically significant. The standard error was used to represent the variation between samples. For root length measurements n=6 (>30 roots per experiment), rosette area measurements n≥15 per replicate, for leaf series and leaf area measurements n=6 plants per replicate and for cell analysis n=3. For qPCR analysis 3 biological and 3 technical replicates were used. In addition to determine SAUR 76 transcript abundance in WT leaves and roots n=7 and a p-value of <0.01 was considered to be statistically significant.

## Results and Discussion

**Figure 1:**
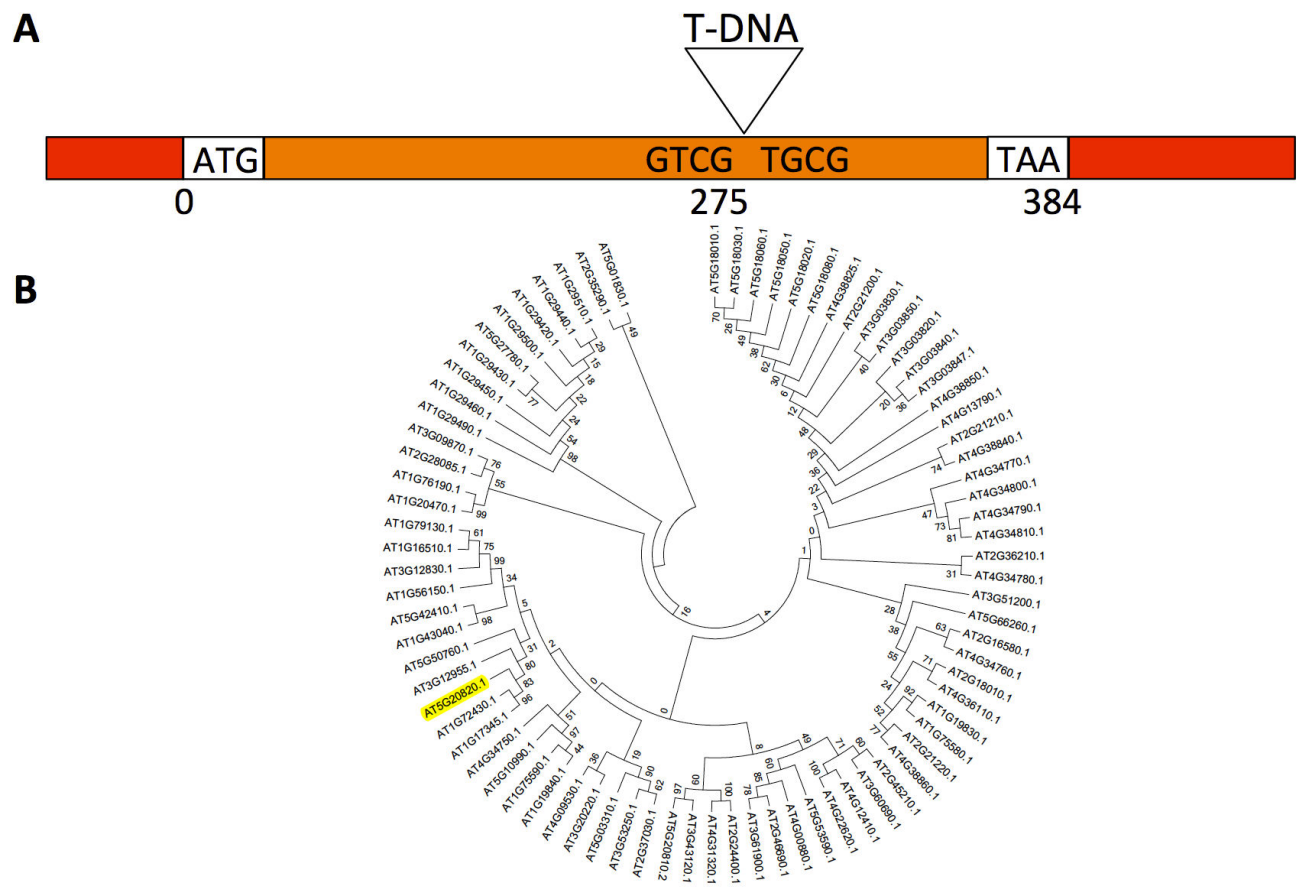
Schematical representation of the SAUR76 gene and its relationship to other members of the SAUR-family. **A**) Linear representation of the SAUR76 gene, including the insertion position of the T-DNA in the knock-out line. Red: UTR; orange: exon; ATG: start codon; TAA: stop codon. **B**) Phylogenetic tree of the Arabidopsis SAUR-family including SAUR-like proteins.

To decipher the mechanism(s) by which ACC mediates the control of cell elongation, a microarray was performed on control and 3h ACC-treated roots. This transcriptome analysis identified 240 differentially expressed genes [26]. Besides genes with a presumptive role in the ethylene-signalling pathway and cell wall metabolism, also several auxin-related genes were identified. Since ethylene is documented to influence auxin transport and biosynthesis [27–30] as part of well documented ethylene/auxin crosstalk [31], this was as expected. In the list of the 10 most upregulated genes by ACC, one predicted auxin-related gene, At5g20820, was present. This gene encodes a one-exon-protein of 127 amino acids (Figure 1A) that is, according to the TAIR website (www.arabidopsis.org), predicted to belong to the SAUR-like auxin-responsive protein family based on the presence of an auxin-responsive SAUR-protein domain (IPR003676).

**Figure 2:**
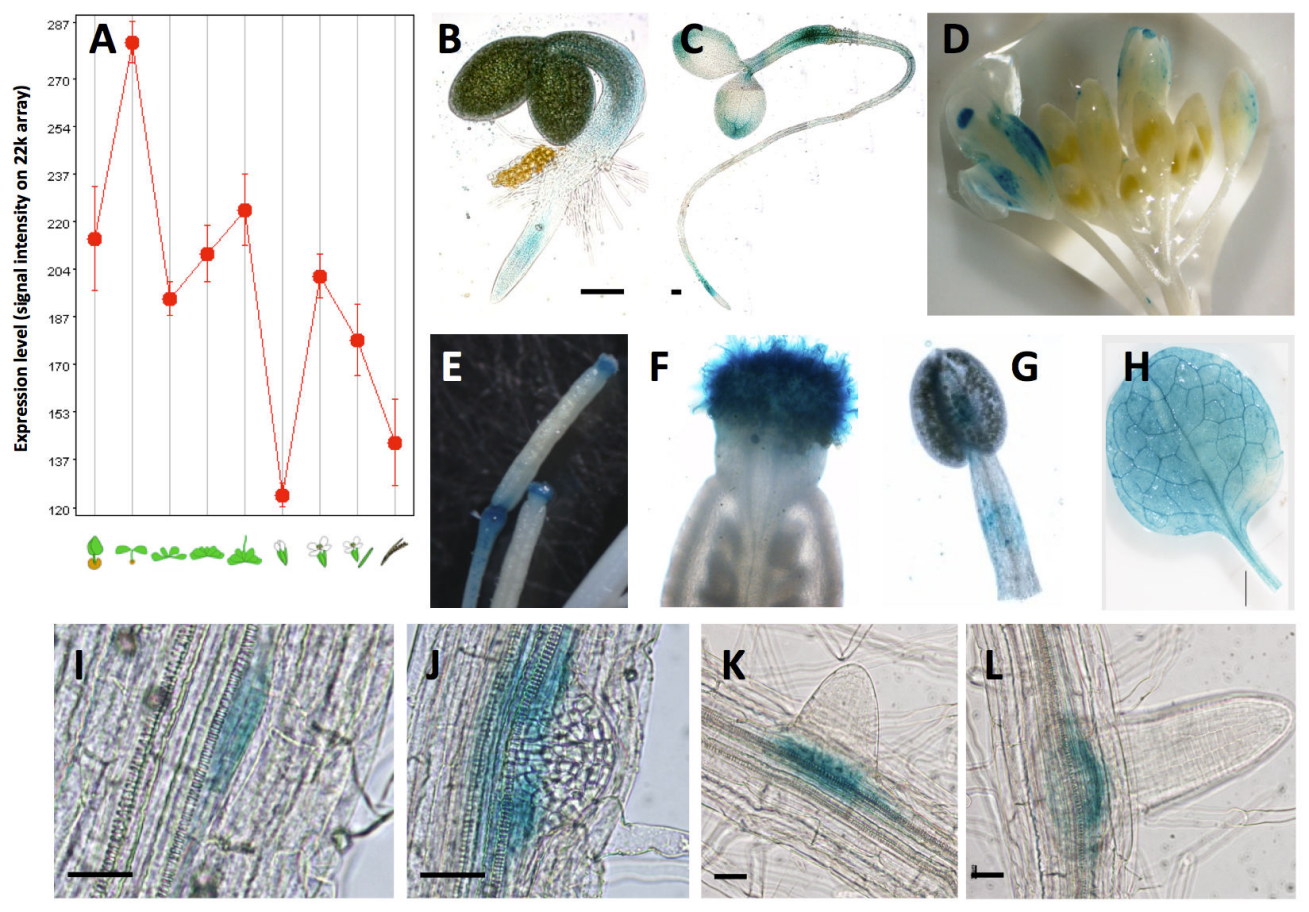
Expression analysis of SAUR76. **A**) In silico expression analysis of SAUR76 using Genevestigator expressed as signal intensities on the 22k array. Expression of SAUR76 seen as GUS activity in promoter-reporter lines, in seedlings (**B**-**C**), flowers (**D**), stamens (**E**-**F**), filaments (**G**), leaves (**H**) and during several phases of lateral root development (**I**-**L**). Scale bar is 100 μm in B and C, 25 μm in I-L.

A protein BLAST was performed to find its closest relatives in Arabidopsis and the genes At1g72430 (SAUR78) and At1g17345 (SAUR77), both members of the SAUR-like auxin-responsive protein family, with similarities of 98% and 99% and identities of 49% and 48% respectively, were found. Furthermore, TAIR identifies 82 genes that code for proteins that belong to the small auxin-up RNAs (SAUR) or SAUR-like family. A phylogenetic tree of the SAUR and SAUR-like family was prepared, and SAUR76 was marked (Figure 1B). In contrast to the trees generated before this study [32], our tree includes SAUR and SAUR-like proteins, which accounts for much more genes, making direct comparison rather difficult. In addition, table S1 presents all TAIR-derived SAUR and SAUR-like genes and also their already used names (with numbers). Further *In silico* analysis with Genevestigator [54] revealed that the expression of SAUR76 is detected throughout plant development and in all organs (Figure 2A). This low-resolution analysis indicates that relatively high expression is found during the early developmental seedling stages of the plant and lower expression in the next stages until the young flower was found. In the developing flower the expression first increases and then decreases until the siliques mature. Confirmation of this in silico analysis was provided by the creation and analysis of 2.1 kb promoter::reporter lines. Several independent lines were investigated, yet only one representative line was used to generate the results shown. Expression in promoter::GUS lines is seen in the cotyledons, hypocotyl and root of young seedlings (Figure 2B-C). GUS activity was also detected in flowers, stamens and filaments (Figure 2D-G), in leaves (Figure 2H) and during several phases of lateral root formation (Figure 2I-L). Reporter-GFP lines confirmed the results (data not shown). 

**Figure 3:**
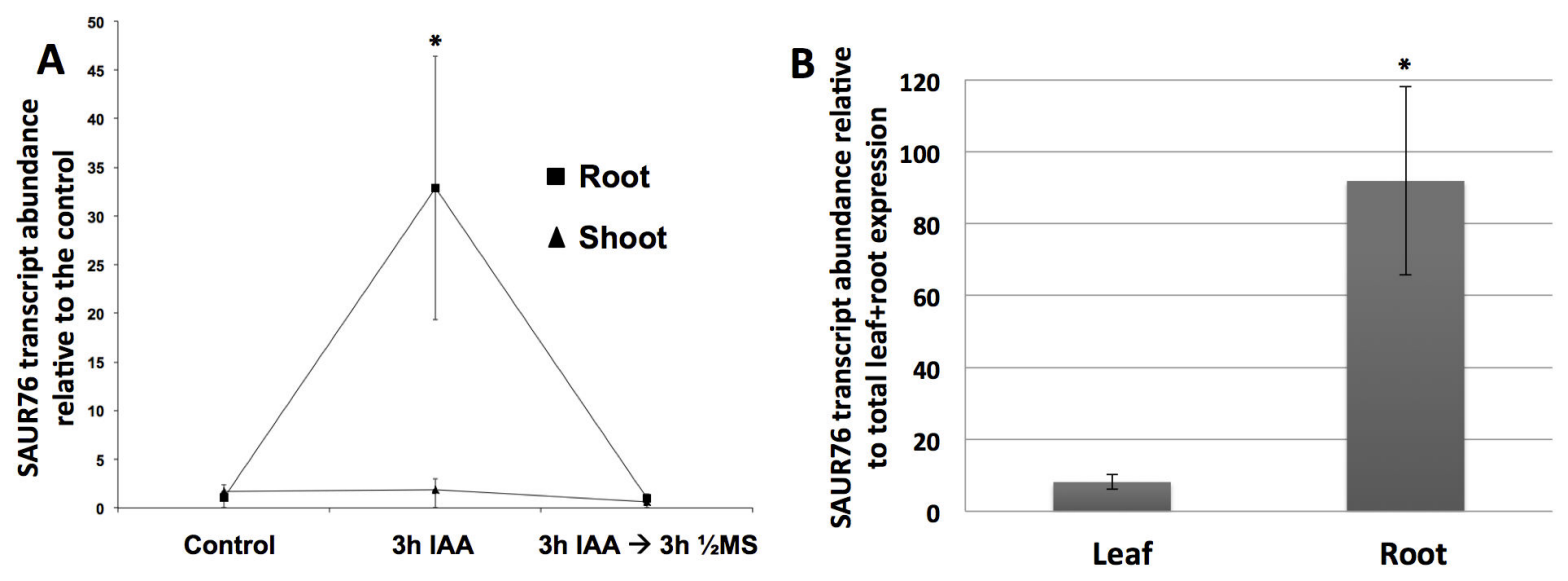
qRT-PCR analysis of SAUR76 expression. **A**) SAUR76 transcript abundance in roots and leaves of control plants, plants treated with 5 μM ACC during 3 hours and of plants that were 3h treated with ACC and re-transferred to control medium for 3 hours. The transcript abundance is expressed relative to the level in control roots or shoots depending on the plant part studied (n=2 replicates, including ≥ 20 roots or ≥ 10 leaves, mean ±an l. **B**) SAUR76 transcript abundance in leaves and roots relative to the total expression in roots and leaves together (n=7, mean ± SE). The asterisks points to a statistical significance towards the control expression level (A) or expression in the leaves (B).

Since SAUR-genes are known to respond rapidly and transiently [33,55] to auxin, seedlings/roots were treated during 3h with IAA after which they were transferred back to control medium. Individual root and leaf samples were taken in control plants, plants treated with IAA and in plants after transfer from the IAA-containing medium to a control medium (without IAA). Relative quantification of SAUR76 expression with qPCR confirmed a strong auxin-induced up regulation of SAUR76, but only in the roots (Figure 3A). In leaves, no significant up regulation was seen. In a control experiment to confirm that auxin was taken up into leaves, auxin was clearly able to induce DR5::GUS in leaves and roots (Figure S1). Three hours after transfer to control medium, however, the expression level of SAUR76 returned to control values in the roots. This auxin-induced up regulation and (fast) return to control levels further supports the idea that SAUR76 indeed belongs to the SAUR family as this is one of the characteristic features [35,36]. Furthermore, analysis of untreated roots and leaves demonstrated that SAUR76 is higher expressed in roots than in leaves (Figure 3B).

**Figure 4:**
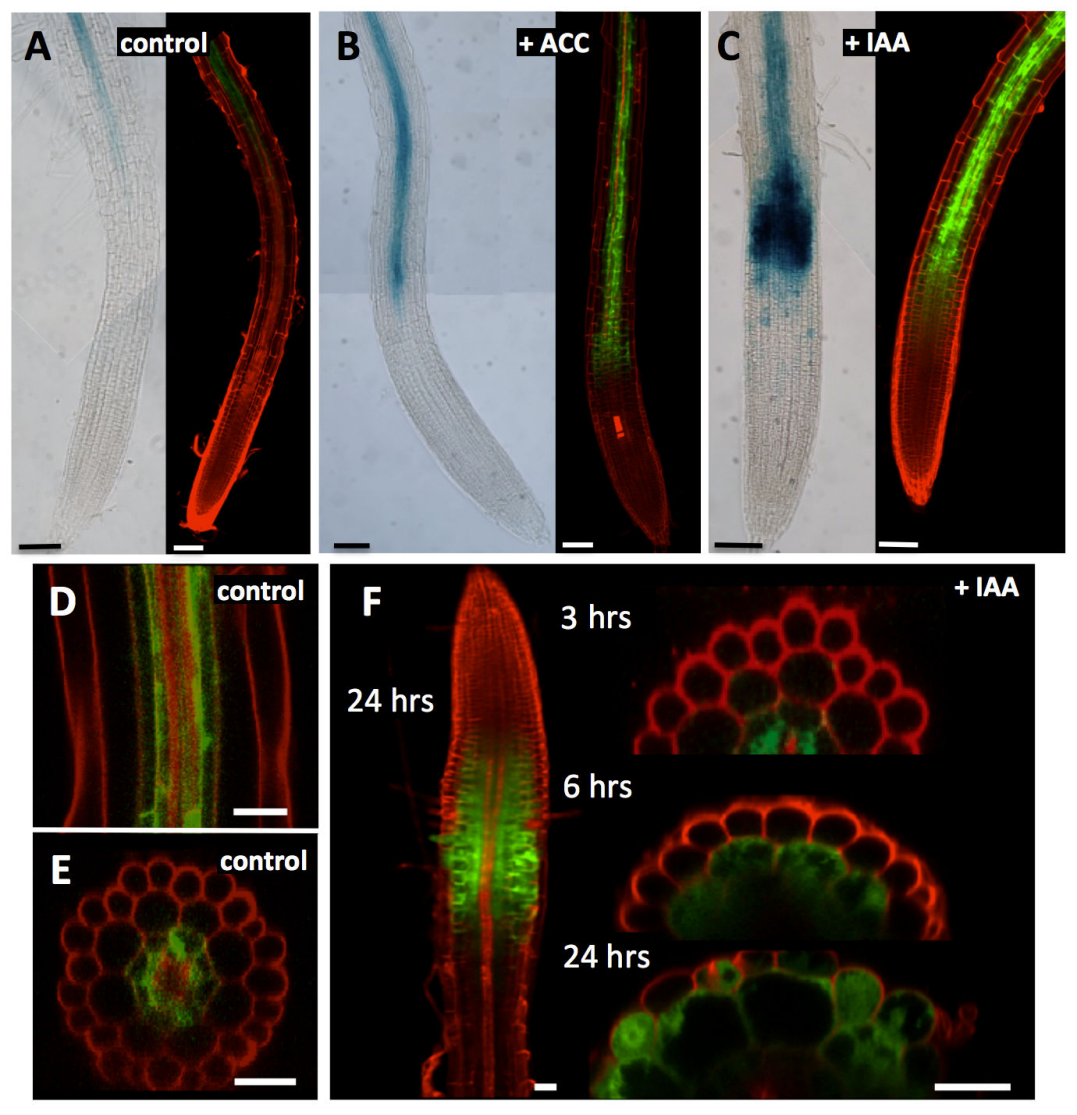
Expression analysis of SAUR76 in control roots and roots treated with ACC and IAA. **A**) Expression of SAUR76 seen in promoter::GUS (left) and promoter::GFP lines (right) in control conditions. **B**) Expression of SAUR76 in roots after 3 h treatment with 5μM ACC or C) 5μM IAA. **D**) Confocal mid-plane longitudinal and **E**) transverse section in the differentiation zone of untreated promotor::GFP roots and **F**) after 3, 6 and 24 hrs treatment with 5μM IAA. Scale bars are 100μm in A-C, 30μm in D-E and 20 μm in F.

A putative promoter sequence upstream of the start codon was used in an in silico analysis using PlantCARE [56] and Athena [57] as resources together with GENEVESTIGATOR [54]. This revealed several auxin-, GA- and methyl jasmonate responsive cis elements (Figure S2), in addition to potential regulation by ABA and paclobutrazol (PAC). The GUS and GFP reporter lines were treated with these hormones to experimentally confirm these *in silico* data. As can be seen from Figure 4, both SAUR76-promoter-driven GUS and GFP-lines reacted in a similar way to the different treatments. In control conditions (Figure 4A), the rather faint expression of At5g20820 is seen in the central part of the root starting from the end of the elongation zone/start of the differentiation zone on. On a confocal mid-plane longitudinal section (Figure 4D) and on a transverse section (Figure 4E) in the differentiation zone of untreated roots the cell types with SAUR76 expression were identified as pericycle and endodermis. Expression was never seen in the outer cell layers. When roots were treated with 5mM ACC during 3 hours, the expression became clearly up regulated (Figure 4B), confirming the finding of the microarray analysis. Furthermore, SAUR76 expression became evident already in cells that had just left the meristematic zone and it appeared in cell layers outside the endodermis. This up regulation was a bit faster and even more pronounced after IAA treatment (Figure 4C), and was confirmed in roots treated with NAA (data not shown). Furthermore, both in SAUR76-promoter::GUS and GFP reporter lines, addition of ABA and PAC resulted in a clear down regulation of At5g20820 expression (data not shown), confirming the *in silico* promoter analysis. After prolonged exposure to IAA, SAUR76 became expressed not only in endodermal cells, but also in cortical cells (after 6 hours), and even in epidermal cells after 24 hours (Figure 4F). This up regulation was confirmed during the process of lateral root formation. When Arabidopsis seedlings were treated during 6 hours with ACC or IAA, higher expression was seen in lateral root primordia than in control conditions (data not shown).

It was shown before that ethylene/ACC acts by regulating IAA transport/biosynthesis [27–30]. The small lag of the expression spreading towards outer cell types and shifting to the start of the elongation zone seen after ACC treatment in comparison with IAA addition is in accordance with this finding. In this case, exogenous addition of IAA/NAA bypasses this ACC-induction of IAA and as a consequence, the response is faster when IAA is added directly. 

**Figure 5:**
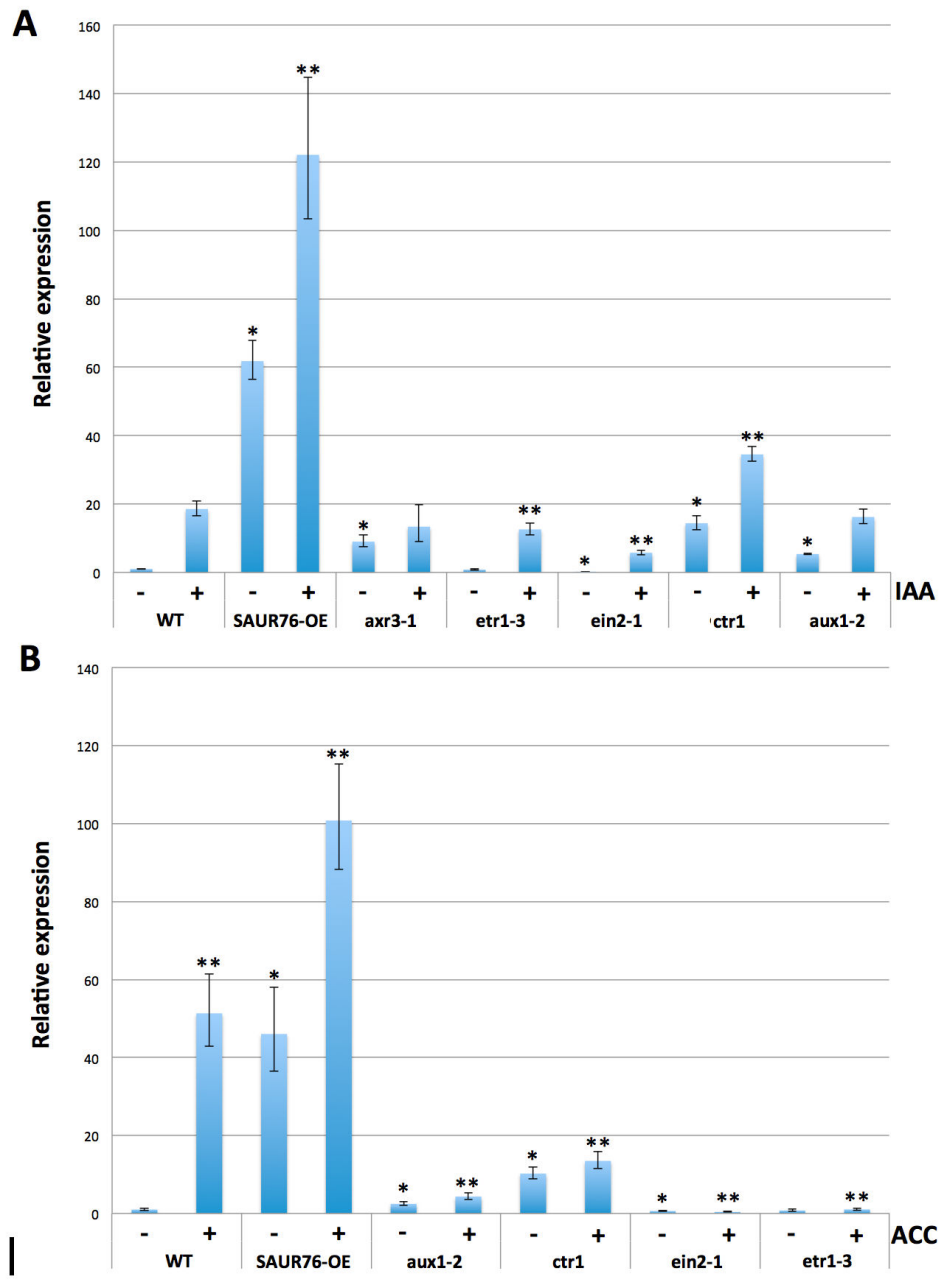
qRT-PCR expression analysis of SAUR76 in ethylene and auxin-mutants in control conditions and after treatment with ACC or auxin. **A**) Effect of 3 hr IAA or B) ACC treatment on SAUR76 expression levels in wild type, ethylene and auxin mutants. * points to a statistical significance towards the untreated WT and ** towards the treated WT (n=3, mean ± SE).

To reveal whether the ACC-induced up regulation of SAUR76 occurred directly by ACC or by the ACC-induced increase in auxin, different mutants in ethylene and auxin signalling were treated with ACC and IAA, and the expression levels of SAUR76 were determined by qPCR (Figure 5). It is known that when ethylene binds to the ETR1 receptor, CTR1 may allow interaction of EIN2 with the kinase domain of ETR1. CTR1 is a negative regulator of ethylene by blocking the signalling downstream to EIN2, so the *ctr1* mutant will have lost the blocking capacity resulting in a constitutive response to ethylene. Both *etr1-*3 and *ein2-1* mutants are insensitive towards ethylene as they cannot sense or transduce the signal respectively (reviewed in 58). Under control conditions, SAUR76 was on average 10 times higher expressed in *ctr1* than in the WT, whereas no significant difference in expression was seen between wild type and the ethylene-insensitive *etr1-3*, while expression of SAUR76 was approximately 40% less in *ein2-1* plants. This agrees with the observed up regulation of SAUR76 in ACC-treated wild type seedlings, as the *ctr1* plants mimic a wild type plant treated with ACC. Upon ACC addition, the increase in expression of SAUR76 was much higher in the WT than in the treated *ctr1* plants, suggesting that efficient and working ethylene perception and signalling is required to increase the expression of SAUR76 (Figure 5A). As expected in the complete ethylene-insensitive *etr1-3* background no ACC-effect on the expression levels was seen, while even a further reduction of the expression was apparent in ACC-treated *ein2-1* plants. In the auxin-import mutant *aux1-2* [59], SAUR76 expression is higher than in the WT under control conditions. SAUR76 expression shows only marginal increase in *aux1-2* after ACC treatment, indicating that AUX1 plays a prominent role in the observed increase of SAUR76 and that maybe the ACC-induced increase of auxin [27–30] is the key-regulator of the observed increases in expression. Indeed, IAA treatments induced a significant up regulation of SAUR76 in all ethylene-related mutants and the WT (Figure 5B), providing further evidence that the increase of SAUR76 by ACC is mostly occurring through the up regulation of auxin signalling. As a consequence, it could be that different auxin levels are present in the *ein2-1* and *aux1-2* mutant, explaining the differences in SAUR76 expression under control and in ACC conditions. Furthermore, *aux1-2* shows an up regulation of SAUR76 expression after IAA treatment. As auxin is not only transported into the cell by AUX1 transporters but also by diffusion through the membrane [60], the bulk addition of auxin in this case can lead to a higher auxin concentration in the cell even when AUX1 function is impaired, and hence an up regulation of SAUR76. Furthermore, the fact that SAUR76 expression levels in untreated *aux1-2* plants is higher than in the wild type and that it is under control of auxin suggests that the endogenous auxin levels might be disturbed in *aux1-2*. This in turn suggests that there is a feedback mechanism between one/several of the auxin signaling components [61] and auxin biosynthesis itself. In addition, the increase in SAUR76 expression in overexpression plants treated with IAA or ACC is higher than in the WT treated with IAA or ACC, providing further evidence that there must be some kind of feedback-mechanism where the SAUR76 protein plays an active role. How exactly this feedback mechanism is regulated, remains to be uncovered. Nevertheless, the outcome of this analysis is that the ACC-induced pathway leading to an increase in expression of SAUR76 goes through changes in auxin concentration. 

**Figure 6:**
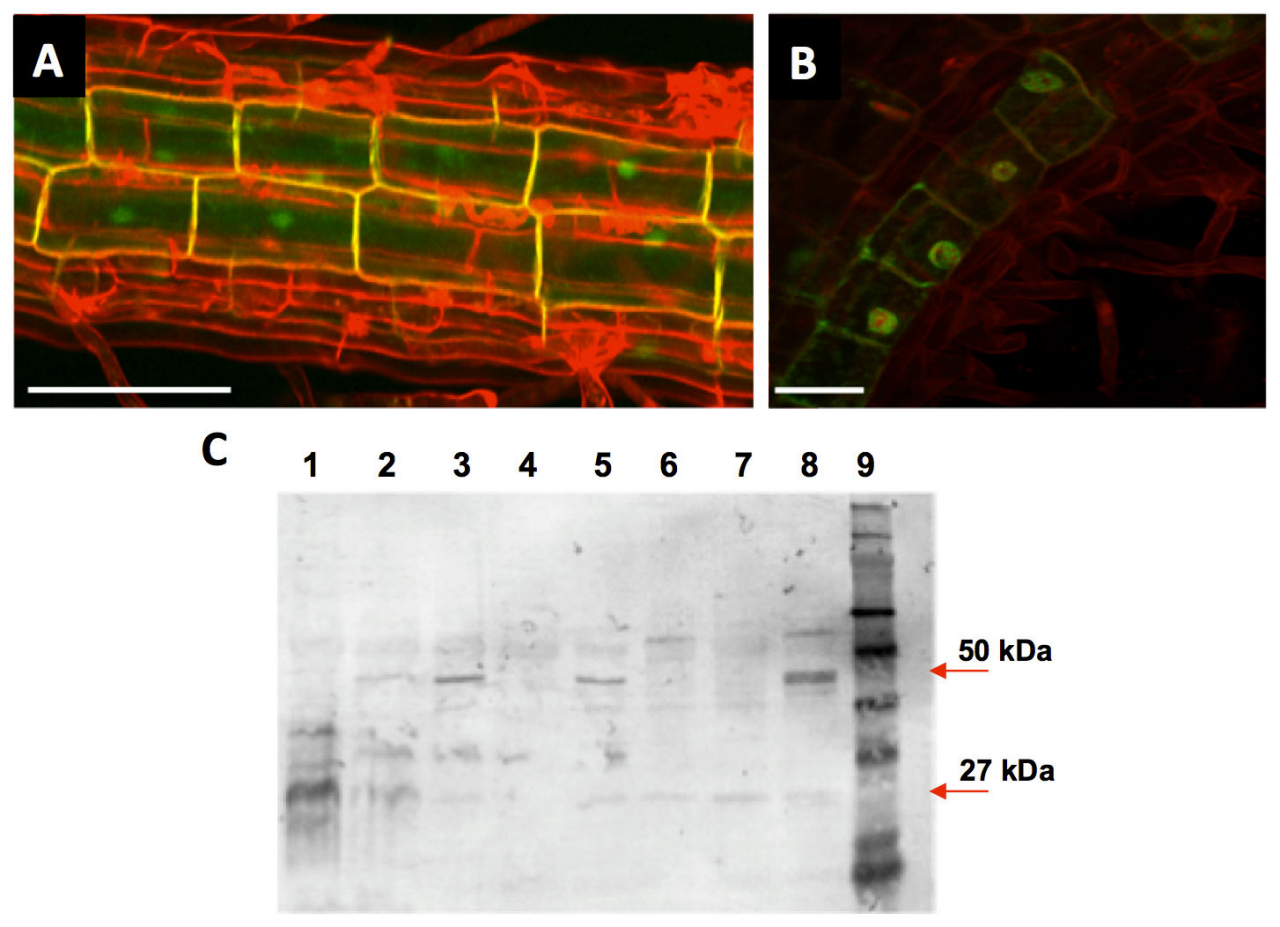
Subcellular location of SAUR76-protein revealed by protein-GFP fusions. **A**) Projected Z-stack image of C-terminal GFP fused to the SAUR76 protein. Cell walls were counterstained with propidium iodide. **B**) Co-localisation of C-terminal GFP fused to the SAUR76 protein and DRAQ5, a red fluorescent nucleus stain. Scale bars are 50 and 25μm in A and B respectively. **C**) Western blot analysis using an anti-GFP antibody on proteins from plants bearing free GFP (lane 1) and from several independent lines containing the C-terminal protein-GFP construct (lane 2-8). Lane 9 contains the molecular marker.

From the 82 putative SAUR or SAUR-like genes, 'Subcellular Location Predictor' indicates that 49 of them are located either in the nucleus or in the cytosol, based on local features of amino acids (sunflower.kuicr.kyoto-u.ac.jp). However, it was shown that SAUR proteins can be plasmamembrane-localised and/or cytosolic [33,34], which is not completely in accordance with the predicted location. The localisation of SAUR76 protein was therefore investigated in transgenic C and N-terminal GFP protein-fusion lines. Figure 6A shows the projected image of a Z-stack through the root with GFP suggested to be present in the cytoplasm, plasma membrane and in the nucleus. To make sure that the observed GFP was indeed also present in the nucleus, a double labelling experiment with DRAQ5, a rapidly staining dye for dsDNA [62], was performed. In Figure 6B an overlap can be seen between the GFP signal and the red signal from DRAQ5, confirming that GFP is indeed located inside the nucleus as well and not only around it and in the plasmamembrane. Furthermore, western blot analysis using an antibody against GFP revealed that part of the GFP seen is indeed fused to the protein, while another part is free GFP (Figure 6C). In lane 1 the proteins of a promoter::GFP line were blotted to indicate the size of free GFP on the blot and the anti-GFP antibody marks free GFP at around 27kDa. Lane 2-8 represent the western blot on different independent SAUR76-GFP lines that were generated in this study and lane 9 contains the molecular size marker. Lane 3, 5 and 8 show a clear band at 50kDa, the estimated size of the protein-GFP fusion. From these different transgenic lines, the plant whose proteins are within lane 8 was used to generate the images in Figure 6A and B. However, lines that show protein-bound GFP also contain a faint band that is visible at 27kDa suggesting that part of the GFP seen on Figures 5A and B is free and not protein-bound. The fact that SAUR76 is present in the nucleus suggests that it might act as a transcription factor, downstream of the ACC/auxin signal, which also confirms the hypothesis of Weijers and Friml [63]. However, given that part of the GFP seen on Figures 5A and B is free, it is possible that the physiological relevant localisation of SAUR76 is the cytoplasm or the plasmamembrane, as was mentioned [33,34]. 

**Figure 7:**
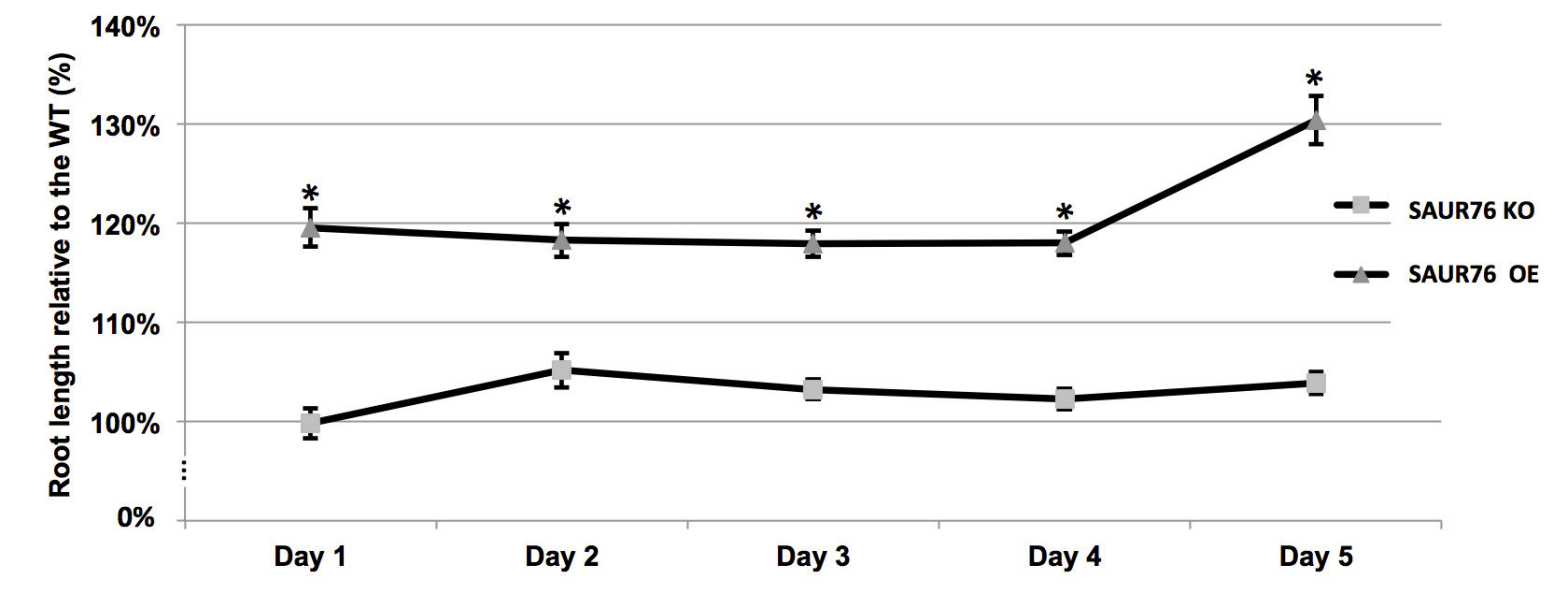
Root growth analysis of wild type, knock-out and SAUR76 overexpressing plants. Plants were grown on 1/2MS medium and root growth was monitored relative to the length of the wild type. The asterisks points to a statistical significance towards the WT (n=6, mean ± SE).

A knock-out (KO) line in SAUR76 was identified and the exact insertion position of the T-DNA insertion was located at approximately 2/3 of the exon using gene and T-DNA specific primers (Figure 1A). Several independent transgenic lines bearing a CaMV 35S-driven overexpression of the gene were generated. Based on semi-transcriptional and qPCR analysis the knock-out was identified as being a null mutant and one overexpression line with the highest expression level was selected for use in root phenotyping experiments. When wild type, knock-out and overexpression lines were grown on 1/2 MS plates, the roots of the overexpression line were always approximately 20% longer than WT and KO. A germination test revealed that a possible difference in germination rate was not responsible for this observed growth phenotype, as all plants germinated at the same time. The difference in root length between the overexpression and wild type seedlings even increased from the 4th day on (Figure 7). No significant difference was seen between root growth of wild type and knock-out plants. The fact that the overexpression of SAUR76 causes longer roots seems contradictory to the finding that the gene becomes up regulated in the ACC or IAA-induced response leading to a shorter root phenotype. These data could lead to a possible conclusion that the gene plays a role in recovery or in controlling the levels of response to stresses caused by auxin or ACC. How exactly it functions remains to be uncovered. Moreover several treatments with different concentrations of hormones, in 5/6-day-old seedlings did not show any clear phenotype, between WT, KO and overexpression plants, when results were normalized to WT. Statistical differences were only observed in the very late stages of the experiments (day 12-15).

**Figure 8:**
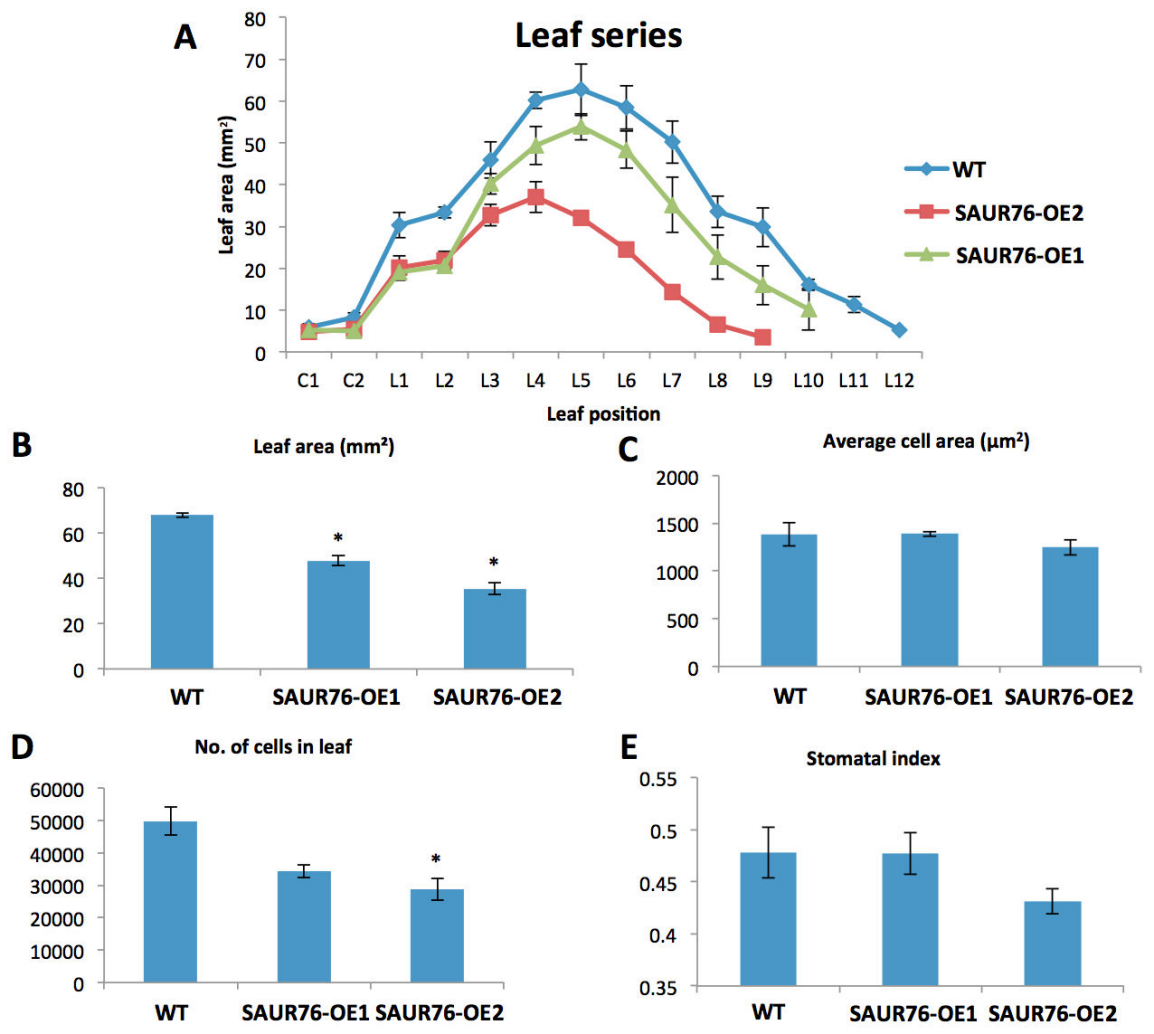
Effect of SAUR76-overexpression on leaf development. **A**) Area of leaf series of the wild type and two independent overexpression lines. **B**) Leaf area of WT and 2 overexpressor lines. **C**) Cell area, **D**) number of cells and **E**) stomatatal index in leaf 4 of WT and 2 overexpressor lines. Asterisks represent statistical differences towards the wild type and results are presented as means ± SE.

A more detailed analysis of the overexpression line revealed that the length of the epidermal cell with the first visible root hair bulge, a measurement for mature cell length [23], was not altered. However, an increased meristem cell number was found, suggesting that the ectopic overexpression of SAUR76 influenced meristem size (WT: 30.7 ± 0.8; overexpression line: 34.8 ± 1.0; p = 0.002; data not shown). As the gene was just moderately expressed in leaves, the effect of the overexpression on leaf development was studied (Figure 8). Leaf series of the wild type and two independent overexpression lines (with different expression levels as revealed by qPCR (WT < SAUR76-OE1 < SAUR76-OE2; data not shown) indicate that the overexpression is negatively correlated with leaf area in a dose-dependent manner (Figure 8A). Leaf 4 was taken for further detailed studies, which indicated that SAUR76 overexpression clearly affects leaf area (Figure 8B) by reducing the number of cells in the leaf and not the size of the cells (Figure 8C and D). No statistical effect was seen on the differentiation as a similar number of stomata were counted (Figure 8E). This again suggests that ectopic expression influences meristematic activity, as was seen in the root. As SAUR76 is induced by auxin, this fits with auxin influencing the cell division process [64]. 

## General Conclusion

SAUR76 is a member of a large family with yet many genes to be characterized. Based on our experiments SAUR76 is present in the nucleus, cytoplasm and plasmamembrane, in agreement with several publications describing some other SAUR genes to be present in the nucleus and plasmamembrane as well [33,34,63,65,66]. SAUR76 plays a minor role in cell elongation somewhere downstream of auxin, and ethylene is influencing the expression of the gene through changes in auxin. Ectopic expression of the gene is capable of influencing meristematic activity, positively in the root, but negatively in the leaf.

## Supporting Information

Table S1 **Table from TAIR showing SAUR and SAUR-like proteins, with inclusion of previously published SAUR-numbers.**
(DOCX)

Figure S1 **Effect of IAA addition to SAUR76 expression in leaves and roots.** IAA was added to the growth medium of promoter-SAUR76::GUS and DR5::GUS plants and GUS activity was assayed in control plants and the IAA-treated plants.
(TIFF)

Figure S2 ***In silico* analysis of SAUR76-promoter sequence.** Cis-elements responsive towards auxin, GA and methyl jasmonate are indicated in the putative promoter sequence upstream of the start codon of SAUR76 using PlantCARE [56] and Athena [57] resources.
(TIFF)
