# Supplementary material for: Characterization of a Small Auxin-Up RNA (SAUR)-Like Gene Involved in Arabidopsis thaliana Development
Source: PLoS One. 2013 Nov 27;8(11):e82596. doi: 10.1371/journal.pone.0082596 (PMC3842426; doi:10.1371/journal.pone.0082596)
Supplement: Table S1 — Table from TAIR showing SAUR and SAUR-like proteins, with inclusion of previously published SAUR-numbers. (DOCX) [file pone.0082596.s001.docx]

| 1 | AT4G34770 | SAUR1 SMALL AUXIN UPREGULATED RNA 1 |
| --- | --- | --- |
| 2 | AT4G34780 | SAUR2 SMALL AUXIN UPREGULATED RNA 2 |
| 3 | AT4G34790 | SAUR3 SMALL AUXIN UPREGULATED RNA 3 |
| 4 | AT4G34800 | SAUR4 SMALL AUXIN UPREGULATED RNA 4 |
| 5 | AT4G34810 | SAUR5 SMALL AUXIN UPREGULATED RNA 5 |
| 6 | AT2G21210 | SAUR6 SMALL AUXIN UPREGULATED RNA 6 |
| 7 | AT2G21200 | SAUR7 SMALL AUXIN UPREGULATED RNA 7 |
| 8 | AT2G16580 | SAUR8 SMALL AUXIN UPREGULATED RNA 8 |
| 9 | AT4G36110 | SAUR9 SMALL AUXIN UPREGULATED RNA 9 |
| 10 | AT2G18010 | SAUR10 SMALL AUXIN UPREGULATED RNA 10 |
| 11 | AT5G66260 | SAUR11 SMALL AUXIN UPREGULATED RNA 11 |
| 12 | AT2G21220 | SAUR12 SMALL AUXIN UPREGULATED RNA 12 |
| 13 | AT4G38825 | SAUR13 SMALL AUXIN UPREGULATED RNA 13 |
| 14 | AT4G38840 | SAUR14 SMALL AUXIN UPREGULATED RNA 14 |
| 15 | AT4G38850 | ARABIDOPSIS COLUMBIA SAUR GENE 1 ARABIDOPSIS THALIANA SMALL AUXIN UPREGULATED 15 ATSAUR15 SAUR-AC1 SAUR15 SAUR_AC1 SMALL AUXIN UP RNA 1 FROM ARABIDOPSIS THALIANA ECOTYPE COLUMBIA SMALL AUXIN UPREGULATED 15 |
| 16 | AT4G38860 | SAUR16 SMALL AUXIN UPREGULATED RNA 16 |
| 17 | AT4G09530 | SAUR17 SMALL AUXIN UPREGULATED RNA 17 |
| 18 | AT3G51200 | SAUR18 SMALL AUXIN UPREGULATED RNA 18 |
| 19 | AT5G18010 | SAUR19 SMALL AUXIN UP RNA 19 |
| 20 | AT5G18020 | SAUR20 SMALL AUXIN UP RNA 20 |
| 21 | AT5G01830 | SAUR21 SMALL AUXIN UP RNA 21 |
| 22 | AT5G18050 | SAUR22 SMALL AUXIN UP RNA 22 |
| 23 | AT5G18060 | SAUR23 SMALL AUXIN UP RNA 23 |
| 24 | AT5G18080 | SAUR24 SMALL AUXIN UP RNA 24 |
| 25 | AT4G13790 | SAUR25 SMALL AUXIN UPREGULATED RNA 25 |
| 26 | AT3G03850 | SAUR26 SMALL AUXIN UP RNA 26 |
| 27 | AT3G03840 | SAUR27 SMALL AUXIN UP RNA 27 |
| 28 | AT3G03830 | SAUR28 SMALL AUXIN UP RNA 28 |
| 29 | AT3G03820 | SAUR29 SMALL AUXIN UP RNA 29 |
| 30 | AT5G53590 | SAUR30 SMALL AUXIN UPREGULATED RNA 30 |
| 31 | AT4G00880 | SAUR31 SMALL AUXIN UPREGULATED RNA 31 |
| 32 | AT2G46690 | SAUR32 SMALL AUXIN UPREGULATED RNA 32 |
| 33 | AT3G61900 | SAUR33 SMALL AUXIN UPREGULATED RNA 33 |
| 34 | AT4G22620 | SAUR34 SMALL AUXIN UPREGULATED RNA 34 |
| 35 | AT4G12410 | SAUR35 SMALL AUXIN UPREGULATED RNA 35 |
| 36 | AT2G45210 | SAG201 SAUR36 SENESCENCE-ASSOCIATED GENE 201 SMALL AUXIN UPREGULATED 36 |
| 37 | AT4G31320 | SAUR37 SMALL AUXIN UPREGULATED RNA 37 |
| 38 | AT2G24400 | SAUR38 SMALL AUXIN UPREGULATED RNA 38 |
| 39 | AT3G43120 | SAUR39 SMALL AUXIN UPREGULATED RNA 39 |
| 40 | AT1G79130 | SAUR40 SMALL AUXIN UPREGULATED 40 |
| 41 | AT1G16510 | SAUR41,SMALL AUXIN UPREGULATED 41 |
| 42 | AT2G28085 | SAUR42 SMALL AUXIN UPREGULATED RNA 42 |
| 43 | AT5G42410 | SAUR43 SMALL AUXIN UPREGULATED RNA 43 |
| 44 | AT5G03310 | SAUR44 SMALL AUXIN UPREGULATED RNA 44 |
| 45 | AT2G36210 | SAUR45 SMALL AUXIN UPREGULATED RNA 45 |
| 46 | AT2G37030 | SAUR46 SMALL AUXIN UPREGULATED RNA 46 |
| 47 | AT3G20220 | SAUR47 SMALL AUXIN UPREGULATED RNA 47 |
| 48 | AT3G09870 | SAUR48 SMALL AUXIN UPREGULATED RNA 48 |
| 49 | AT4G34750 | SAUR49 SMALL AUXIN UPREGULATED RNA 49 |
| 50 | AT4G34760 | SAUR50 SMALL AUXIN UPREGULATED RNA 50 |
| 51 | AT1G75580 | SAUR51 SMALL AUXIN UPREGULATED RNA 51 |
| 52 | AT1G75590 | SAUR52 SMALL AUXIN UPREGULATED RNA 52 |
| 53 | AT1G19840 | SAUR53 SMALL AUXIN UPREGULATED RNA 53 |
| 54 | AT1G19830 | SAUR54 SMALL AUXIN UPREGULATED RNA 54 |
| 55 | AT5G50760 | SAUR55 SMALL AUXIN UPREGULATED RNA 55 |
| 56 | AT1G76190 | SAUR56 SMALL AUXIN UPREGULATED RNA 56 |
| 57 | AT3G53250 | SAUR57 SMALL AUXIN UPREGULATED RNA 57 |
| 58 | AT1G43040 | SAUR58 SMALL AUXIN UPREGULATED RNA 58 |
| 59 | AT3G60690 | SAUR59 SMALL AUXIN UPREGULATED RNA 59 |
| 60 | AT1G20470 | SAUR60 SMALL AUXIN UPREGULATED RNA 60 |
| 61 | AT1G29420 | SAUR61 SMALL AUXIN UPREGULATED RNA 61 |
| 62 | AT1G29430 | SAUR62 SMALL AUXIN UPREGULATED RNA 62 |
| 63 | AT1G29440 | SAUR63 SMALL AUXIN UP RNA 63 |
| 64 | AT1G29450 | SAUR64 SMALL AUXIN UPREGULATED RNA 64 |
| 65 | AT1G29460 | SAUR65 SMALL AUXIN UPREGULATED RNA 65 |
| 66 | AT1G29500 | SAUR66 SMALL AUXIN UPREGULATED RNA 66 |
| 67 | AT1G29510 | SAUR67 SMALL AUXIN UPREGULATED RNA 67 |
| 68 | AT1G29490 | SAUR68 SMALL AUXIN UPREGULATED 68 |
| 69 | AT5G10990 | SAUR69 SMALL AUXIN UPREGULATED RNA 69 |
| 70 | AT5G20810 | SAUR70 SMALL AUXIN UPREGULATED RNA 70 |
| 71 | AT1G56150 | SAUR71 SMALL AUXIN UPREGULATED 71 |
| 72 | AT3G12830 | SAUR72 SMALL AUXIN UPREGULATED 72 |
| 73 | AT3G03847 | SAUR73 SMALL AUXIN UPREGULATED RNA 73 |
| 74 | AT3G12955 | SAUR74 SMALL AUXIN UPREGULATED RNA 74 |
| 75 | AT5G27780 | SAUR75 SMALL AUXIN UP RNA 75 |
| 76 | AT5G20820 | SAUR76 SMALL AUXIN UPREGULATED RNA 76 |
| 77 | AT1G17345 | SAUR77 SMALL AUXIN UPREGULATED RNA 77 |
| 78 | AT1G72430 | SAUR78 SMALL AUXIN UPREGULATED RNA 78 |
| 79 | AT2G35290 | SAUR79 SMALL AUXIN UPREGULATED RNA 79 |
| 80 | AT5G18030 | SAUR-like auxin-responsive protein family doesn’t have nr? |
