# Supplementary figures and images for: Characterization of a Small Auxin-Up RNA (SAUR)-Like Gene Involved in Arabidopsis thaliana Development
Source: PLoS One. 2013 Nov 27;8(11):e82596. doi: 10.1371/journal.pone.0082596 (PMC3842426; doi:10.1371/journal.pone.0082596)

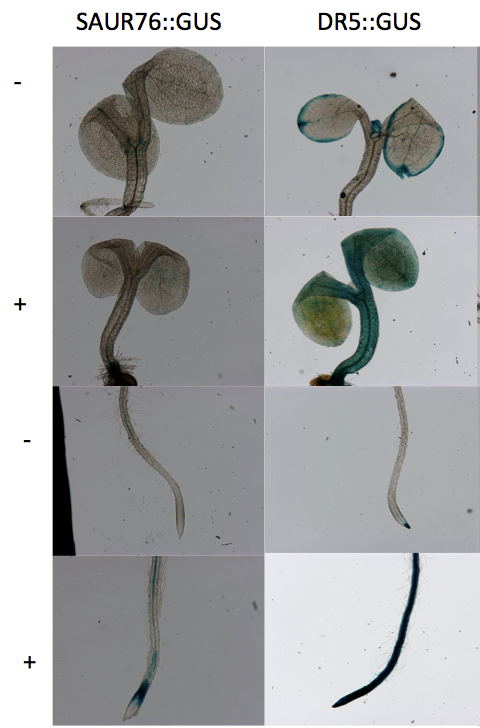

Supplement: Figure S1 — Effect of IAA addition to SAUR76 expression in leaves and roots. IAA was added to the growth medium of promoter-SAUR76::GUS and DR5::GUS plants and GUS activity was assayed in control plants and the IAA-treated plants. (TIFF) [file pone.0082596.s002.tiff]

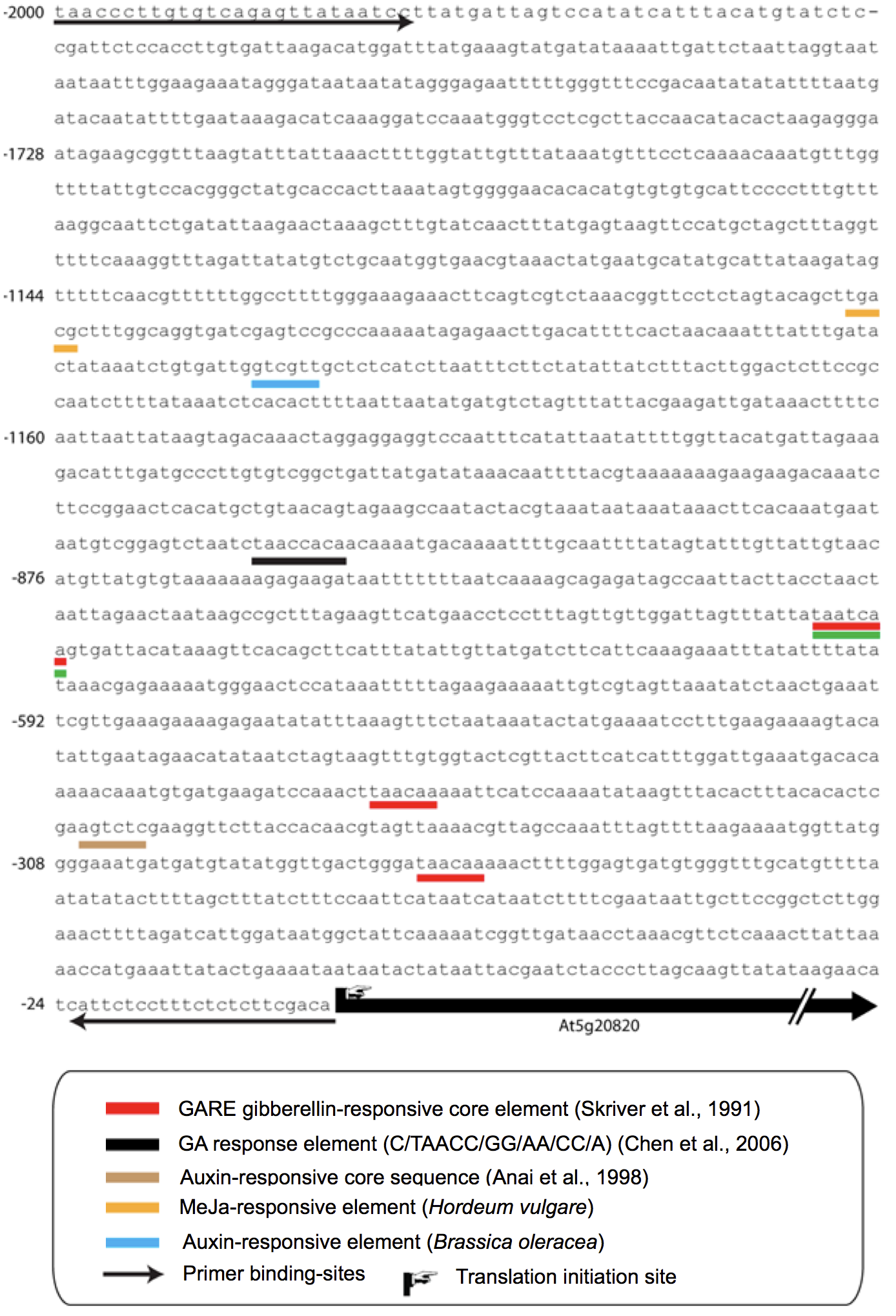

Supplement: Figure S2 — In silico analysis of SAUR76-promoter sequence. Cis-elements responsive towards auxin, GA and methyl jasmonate are indicated in the putative promoter sequence upstream of the start codon of SAUR76 using PlantCARE [56] and Athena [57] resources. (TIFF) [file pone.0082596.s003.tiff]
